# A Potent Antagonist of Smoothened in Hedgehog Signaling for Epilepsy

**DOI:** 10.3390/ijms232314505

**Published:** 2022-11-22

**Authors:** Junwan Fan, Zichen Zhao, Ru Liu, Haowen Li, Wenyan He, Jianping Wu, Yongjun Wang, Wei Chen

**Affiliations:** 1 China National Clinical Research Center for Neurological Diseases, Beijing Tiantan Hospital, Capital Medical University, Beijing 100070, China; 2 Advanced Innovation Center for Human Brain Protection, Capital Medical University, Beijing 100070, China; 3 Beijing Key Laboratory of Translational Medicine for Cerebrovascular Disease, Beijing 100070, China; 4 School of Chemistry, Chemical Engineering and Life Sciences, Wuhan University of Technology, Wuhan 430070, China

**Keywords:** TT22, GDC-0449, LDE-225, hedgehog signaling, smoothened, epilepsy

## Abstract

Epilepsy is one of the common encephalopathies caused by sudden abnormal discharges of neurons in the brain. About 30% of patients with epilepsy are insensitive and refractory to existing antiseizure medications. The sonic hedgehog signaling pathway is essential to the development and homeostasis of brain. Aberrant sonic hedgehog signaling is increased in refractory epileptic lesions and may involve the etiology of epilepsy. Thus, new inhibitors of Smoothened, a key signal transducer of this signaling pathway are urgently need for refractory epilepsy. We have established a high-throughput screening platform and discovered several active small molecules targeting Smoothened including TT22. Here we show that the novel Smoothened inhibitor TT22 could block the translocation of βarrestin2-GFP to Smoothened, reduce the accumulation of Smoothened on primary cilia, displace Bodipy-cyclopamine binding to Smoothened, and inhibit the expression of downstream Gli transcription factor. Moreover, TT22 inhibits the abnormal seizure-like activity in neurons. Furthermore, we demonstrated that FDA-approved Smoothened inhibitor GDC-0449 and LDE-225 are able to inhibit abnormal seizure-like activity in neurons. Thus, our study suggests that targeting the sonic hedgehog signaling with new small-molecule Smoothened inhibitors might provide a potential new therapeutic avenue for refractory epilepsy.

## 1. Introduction

The evolutionarily conserved sonic hedgehog (Shh) signaling pathways play an important role in the development and homeostasis of the central nervous system and Smoothened (Smo) protein is an essential receptor in the Shh signaling pathway [1,2]. The Shh pathway is initiated by the binding of Shh ligands to the twelve-transmembrane receptor patched on the cell membrane, releasing its inhibitory effect on the seven-transmembrane Smo receptor. Activated Smo initiates the translocation of downstream Gli transcription factor to the nucleus and regulates target gene expression [3,4]. It has been reported that the abnormal activation of Shh signaling pathway plays an important role in the development of many types of cancers, including basal cell carcinoma (BCC), medulloblastoma (MB), and other solid tumors [5,6]. Smo antagonists GDC-0449/vismodegib and LDE-225/sonidegib were approved as drugs for BCC and MB in 2012 and 2015, respectively [7,8,9].

Our previous studies have shown that constitutively activated Smo has the ability to recruit β-arrrestin2 proteins to the plasma membrane [2]. Furthermore, coexpression of Smo-633, a chimeric form of Smo swapping the C terminus of Smo with vasopressin type 2 receptor, with βarrrestin2-GFP (βarr2-GFP) in cells resulted in the redistribution of βarr2-GFP to intracellular vesicles as aggregates [10]. Smo antagonist cyclopamine could reverse this phenomenon [10,11,12]. Based on these findings, we constructed a cell-based high-throughput screening assay and screened our propriety small molecules library (chemical scaffold: 3-bromo-4-chlorobenzoic acid) for Smo inhibitors. Several active hits including TT22 were discovered. TT22 can block the translocation of βarr2-GFP to Smo, displace Bodipy-cyclopamine to bind to Smo, reduce the accumulation of Smo on primary cilia, and inhibit Gli1 transcription and expression.

Epilepsy is one of the most common severe encephalopathies, affecting about 70 million people worldwide [13]. About 30% of patients with epilepsy are insensitive and refractory to existing antiseizure medications (ASMs). In addition, patients treated with ASMs often have severe side effects, such as neuropsychiatric symptoms, teratogenicity, and metabolic disturbances. Thus, new small molecule drugs are urgently needed for refractory epilepsy [14,15]. Temporal lobe epilepsy is the most common type of epilepsy that is insensitive and refractory to epilepsy drugs [16,17]. Currently, surgery is the only treatment for patients with refractory epilepsy to control the frequency and degree of epilepsy [18,19].

Previous reports have demonstrated that the expression of Shh increases in the epileptogenic zone of patients and animal models with refractory temporal lobe epilepsy, suggesting that Shh could play an important role in the occurrence and development of refractory epilepsy and provide a new underlying etiology for refractory temporal lobe epilepsy [20,21,22,23]. Furthermore, a recent study has demonstrated that epileptiform discharge induces the increase of Shh from the temporal neocortex, which trigger the Shh release and enhance abnormal neuronal activity leading to the progression of epilepsy [24].

Here we show that TT22 is a novel Smo inhibitor by blocking the translocation of βarr2-GFP to Smo, reducing the accumulation of Smo on primary cilia, displacing Bodipy-cyclopamine binding to Smo, and inhibiting the expression of downstream Gli transcription factor. Moreover, novel TT22 along with clinically approved Smo inhibitors (GDC-0449/vismodegib and LDE-225/sonidegib) inhibit the abnormal seizure-like activity in neurons. These novel findings may have potential as a new therapy targeting Shh signaling for refractory epilepsy.

## 2. Results

### 2.1. Identification of the Compound TT22 as a Smo Inhibitor

**Figure 1:**
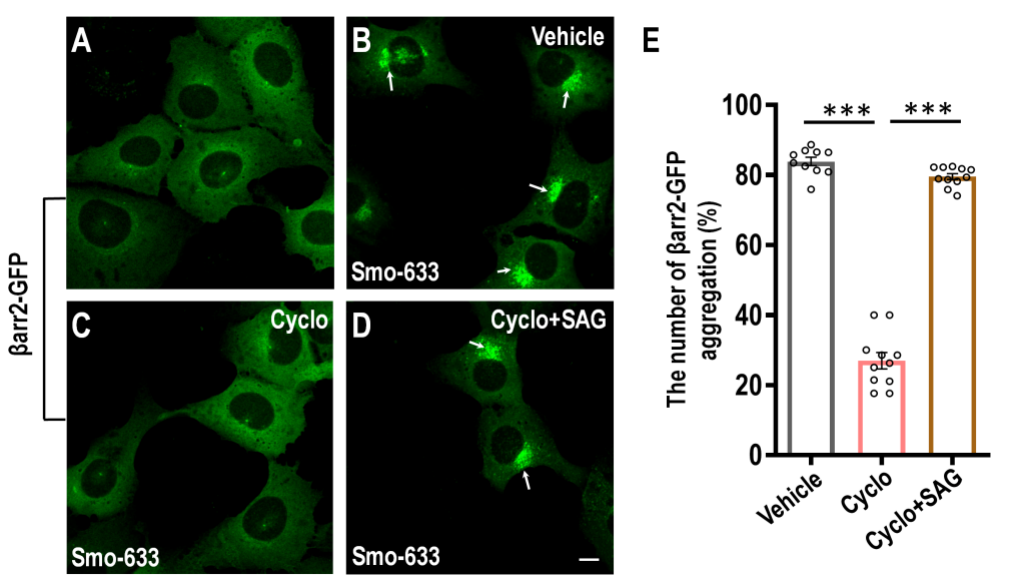
Establishment of the cell-based high-throughput screening assay and identification of novel Smo inhibitors. (**A**–**D**) The U2OS cells stably overexpressing βarr2-GFP alone (**A**); or with βarr2-GFP coexpressed with Smo-633 (**B**–**D**). Cells were treated for 24 h at 37 °C with DMSO vehicle (**B**), with 1 μM cyclopamine (Cyclo) (**C**), or with 1 μM Cyclo and 1 μM SAG (**D**). The arrows show the intravesicular aggregation of βarr2-GFP. (**E**) The percentage of the number of βarr2-GFP aggregation was quantitated. The scale bar is 10 µm. All data are means ± SEM (one-way ANOVA). *** *p* < 0.001.

When βarr2-GFP was expressed in cells alone, it was uniformly distributed throughout the cytoplasm (Figure 1A). Coexpression of βarr2-GFP and Smo-633 led to the complex formation of βarr2-GFP and Smo and redistributed in the intracellular vesicles as aggregates around the nucleus (Figure 1B). At a cyclopamine concentration of 1 μM (a well-known Smo antagonist), the activity of Smo was inhibited and the phenomenon of green fluorescence aggregation of βarr2-GFP could be redistributed in a homogeneous form (Figure 1C). The aggregation of βarr2-GFP could be restored again when cyclopamine and a well-known Smo agonist SAG were present at the same time (Figure 1D). The green aggregates are quantitated in Figure 1E.

**Figure 2:**
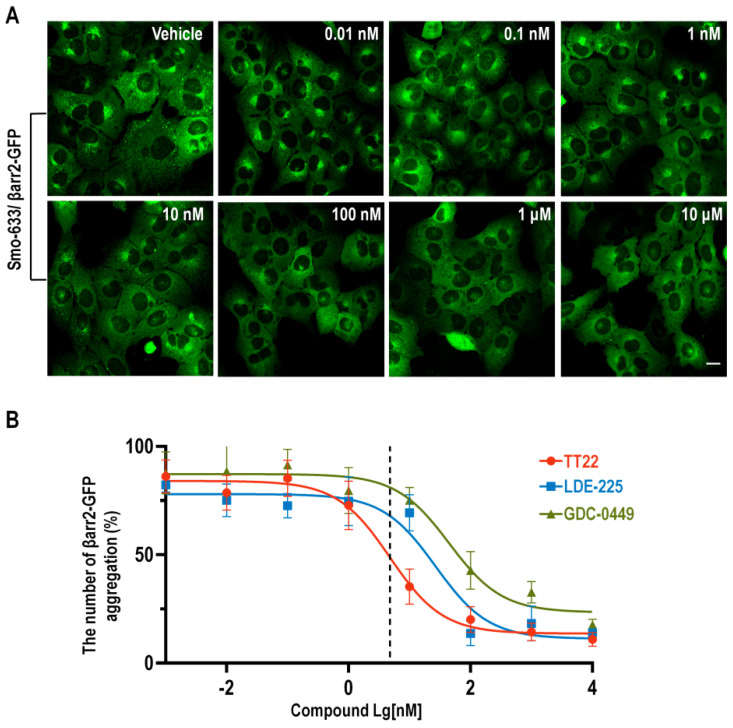
The intracellular aggregation of βarr2-GFP was inhibited by TT22. (**A**) The U2OS cells stably expressing βarr2-GFP and Smo-633 were treated with the indicated concentration of TT22 (10^−12^–10^−5^ M) for 24 h at 37 °C. (**B**) The percentage of the number of βarr2-GFP aggregation was quantitated. The scale bar is 20 µm.

Accordingly, we established a high-throughput screening method for Smo antagonists, screened a small molecule compound library and discovered that several small molecules including TT22 could inhibit the formation of intracellular βarr2-GFP aggregates (Figure 2A). The IC_50_ of TT22, LDE-225 and GDC-0449 were 4.75 nM, 26.78 nM, 44.55 nM, respectively (Figure 2B). 

**Figure 3:**
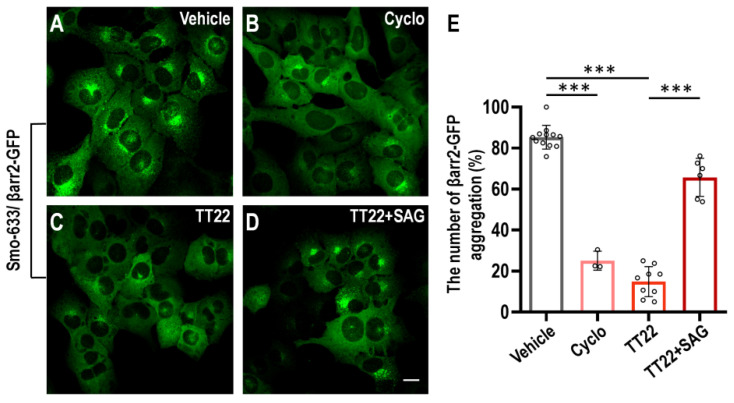
Novel Smo inhibitors were identified in U2OS cells. (**A**–**D**) The U2OS cells stably overexpressing βarr2-GFP and Smo-633 were treated with DMSO vehicle (**A**), 1 μM cyclopamine (Cyclo) (**B**), 100 nM TT22 (**C**), or 100 nM TT22 and 1 μM SAG (**D**) for 24 h at 37 °C. The arrows show the intravesicular aggregation of βarr2-GFP. (**E**) The percentage of the number of βarr2-GFP aggregation was quantitated. The scale bar is 20 µm. All data are means ± SEM (one-way ANOVA). *** *p* < 0.001.

**Figure 4:**
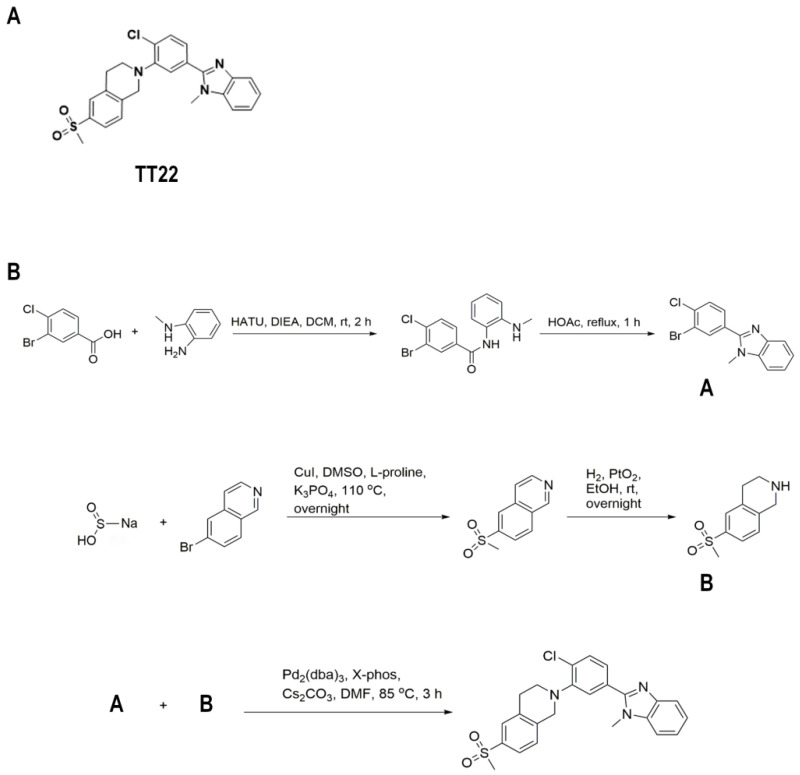
The structure and synthetic route of compound TT22. (**A**) Structure of compound TT22. (**B**) Synthetic route of compound TT22.

To further characterize TT22, we demonstrated that TT22 can block the translocation of βarr2-GFP using cyclopamine as a control (Figure 3A–C). The uniform distribution of βarr2-GFP induced by TT22 in cytoplasm could be reversed by SAG, which further confirmed the TT22 as a Smo inhibitor (Figure 3D). The structure of TT22 is shown in Figure 4A. It was synthesized through the route described in Figure 4B.

### 2.2. TT22 Is a Competitive Antagonist of Smo

**Figure 5:**
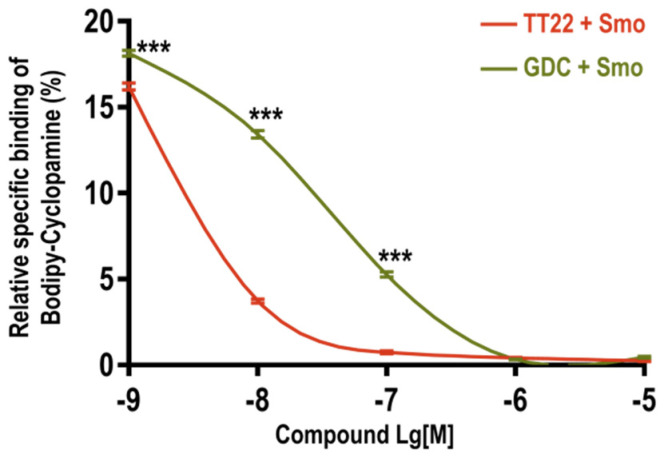
TT22 competes with Bodipy-cyclopamine to bind with Smo. Competitive binding of Bodipy-cyclopamine to Smo with different concentrations (10^−9^–10^−5 M)^ of Smo antagonists: TT22, GDC-0449 (GDC) in HEK293 cells transiently transfected with human wild-type Smo. The binding of Bodipy-cyclopamine (green) was detected by flow cytometry. All data are means ± SEM (two-way ANOVA). *** *p* < 0.001.

We investigated whether TT22 could compete with Bodipy-cyclopamine, which has been used to assess the binding affinity of Smo ligand, to bind Smo [25]. We found that the known Smo antagonists GDC-0449 and TT22 could block the binding of 5 nM Bodipy-cyclopamine to Smo in a dose-dependent manner (Figure 5). These results indicate that TT22 can compete with Bodipy-cyclopamine to bind to the Smo receptor.

### 2.3. TT22 Blocks the Aggregation of Smo on the Primary Cilia

**Figure 6:**
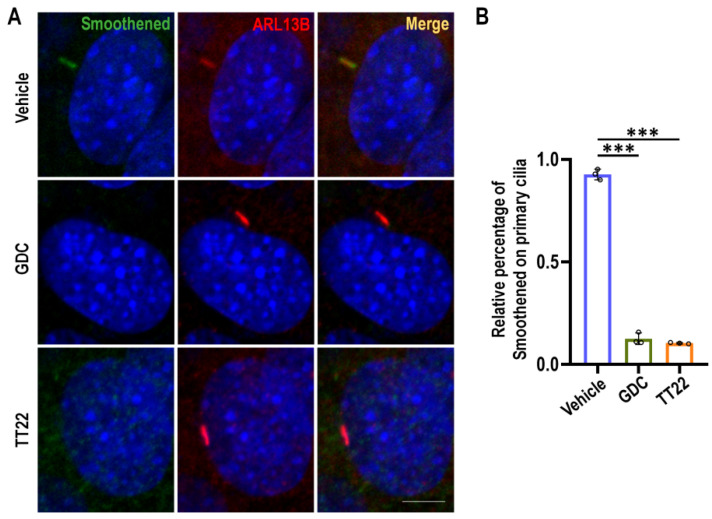
TT22 disrupts the Hh induced Smo accumulation on primary cilia. (**A**) Serum-starved PTCH^−/−^ MEF cells were treated for 24 h at 37 °C with DMSO vehicle, 5 μM TT22, and 5 μM GDC-0449 (GDC). Then, the cells were stained with antibodies against Smoothened (green) and ARL13B (red), a marker for primary cilia, and 5 μM GDC was used as a positive control. (**B**) The percentage of Smo location on primary cilia was quantitated (*n* = 100). The statistical data represented the relative percentage of Smo location on primary cilia between different treatments. The scale bar is 5 µm. All data are means ± SEM (one-way ANOVA). *** *p* < 0.001.

One of the key steps to activate the Shh pathway is the localization and aggregation of Smo on the primary cilia, so we evaluated the effect of TT22 on the localization of Smo using methods as described previously [26,27,28]. We used DMSO, 5 μM GDC-0449, and 5 μM TT22 treated PTCH^−/−^MEF cells for 24 h, and then immunofluorescence staining with anti-Smoothened and ARL13B antibodies. As expected, compared with the DMSO control group, TT22 and GDC-0449 could inhibit the aggregation of Smo on the primary cilia (Figure 6). 

### 2.4. TT22 Inhibits Shh Signaling

**Figure 7:**
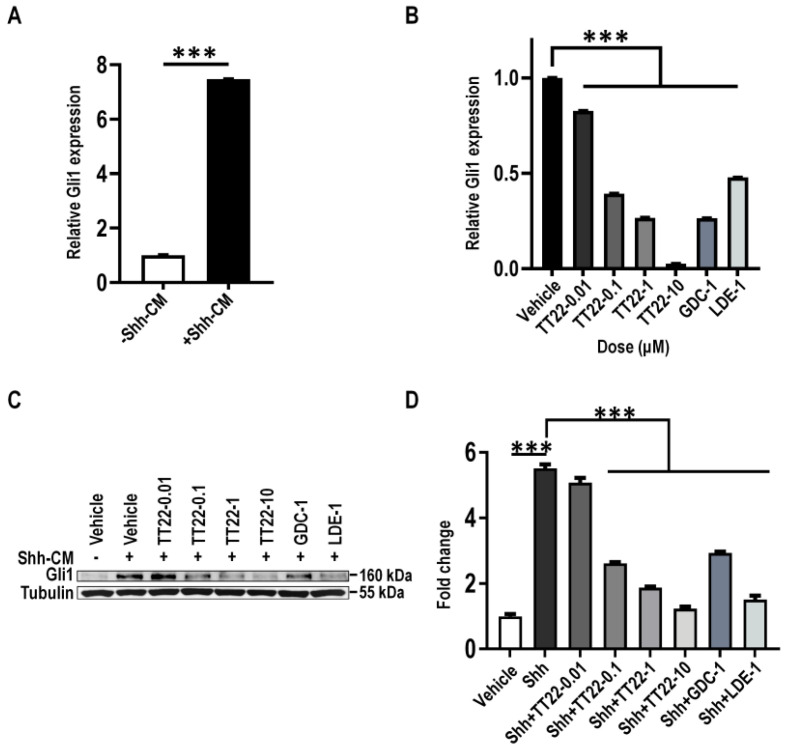
The expression of Gli1 stimulated with Shh-CM was suppressed by TT22. (**A**) Serum-starved NIH3T3 cells were treated with Shh-CM for 24 h, then analyzed for mRNA levels of Gli1. (**B**,**C**) Serum-starved NIH3T3 cells were induced by Shh-CM with DMSO (vehicle), with an indicated concentration of TT22, 1 μM LDE-225 (LDE-1) or 1 μM GDC-0449 (GDC-1) for 24 h. Cells were analyzed for mRNA levels (**B**) and the protein levels (**C**) of Gli1. (**D**) Quantitation of Gli1 protein levels normalized to Tubulin loading control measured the dose-dependent inhibition of the Hh pathway activity by TT22 upon Shh stimulation. All data are means ± SEM (Student’s *t*-test and one-way ANOVA). *** *p* < 0.001.

**Figure 8:**
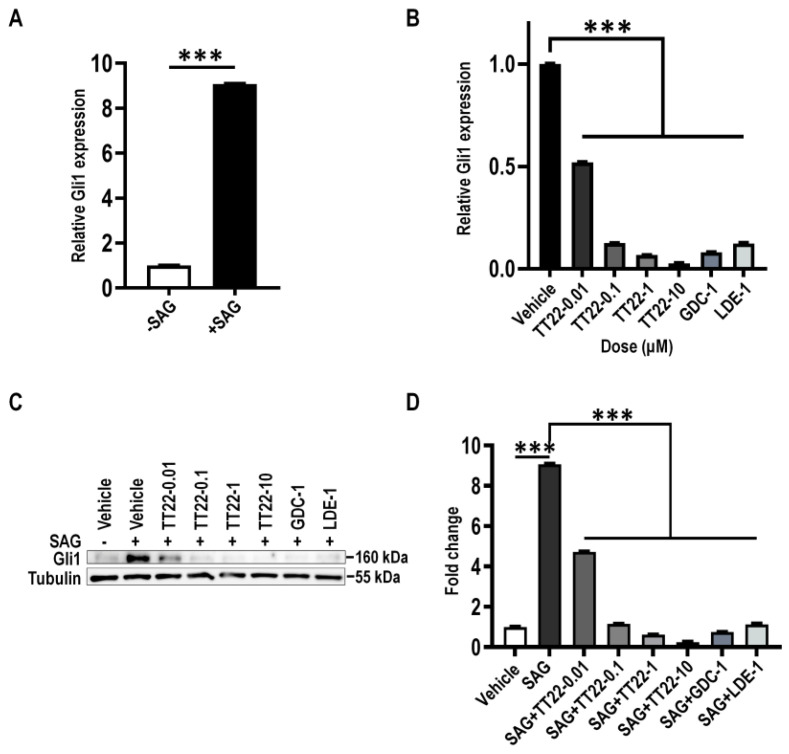
The expression of Gli1 stimulated with SAG was suppressed by TT22. (**A**) Serum-starved NIH3T3 cells were treated with 100 nM SAG for 24 h, then analyzed for mRNA levels of Gli1. (**B**,**C**) Serum-starved NIH3T3 cells were induced by 10^−7^ M (100 nM) SAG with DMSO (vehicle), with the indicated concentration of TT22, 1 μM LDE-225 (LDE-1) or 1 μM GDC-0449 (GDC-1) for 24 h. Cells were analyzed for mRNA levels (**B**) and the protein levels (**C**) of Gli1. (**D**) Quantitation of Gli1 protein levels normalized to Tubulin loading control measured the dose-dependent inhibition of the Hh pathway activity by TT22 upon SAG stimulation. All data are means ± SEM (Student’s *t*-test and one-way ANOVA). *** *p* < 0.001.

Gli1 is the downstream target gene of the Shh signaling pathway [29]. In order to assess the inhibitory effect of TT22 on the downstream of Shh, NIH3T3 cells were treated with *N-Shh* conditioned medium (Shh-CM) or Smo agonist SAG to stimulate the expression of Gli1. We firstly prepared Shh-CM and explored the optimal effective concentration of Shh-CM and SAG for Gli expression through a gradient concentration treatment, which was 20% of CM and 100 nM SAG (Figure 7A and Figure 8A). We observed that 20% Shh-CM or 100 nM SAG stimulated NIH3T3 cells treated with TT22 (0.01, 0.1, 1, and 10 μM) or Smo antagonist GDC-0449 (1 μM), and LDE-225 (1 μM) led to the reduction of the mRNA or protein expression of Gli1 in a dose-dependent manner (Figure 7B–D and Figure 8B–D). These data suggest that TT22 is a novel Smo inhibitor to suppress the expression of Gli. 

### 2.5. TT22 Inhibits the Abnormal Seizure-like Activity in Cultured Neurons 

**Figure 9:**
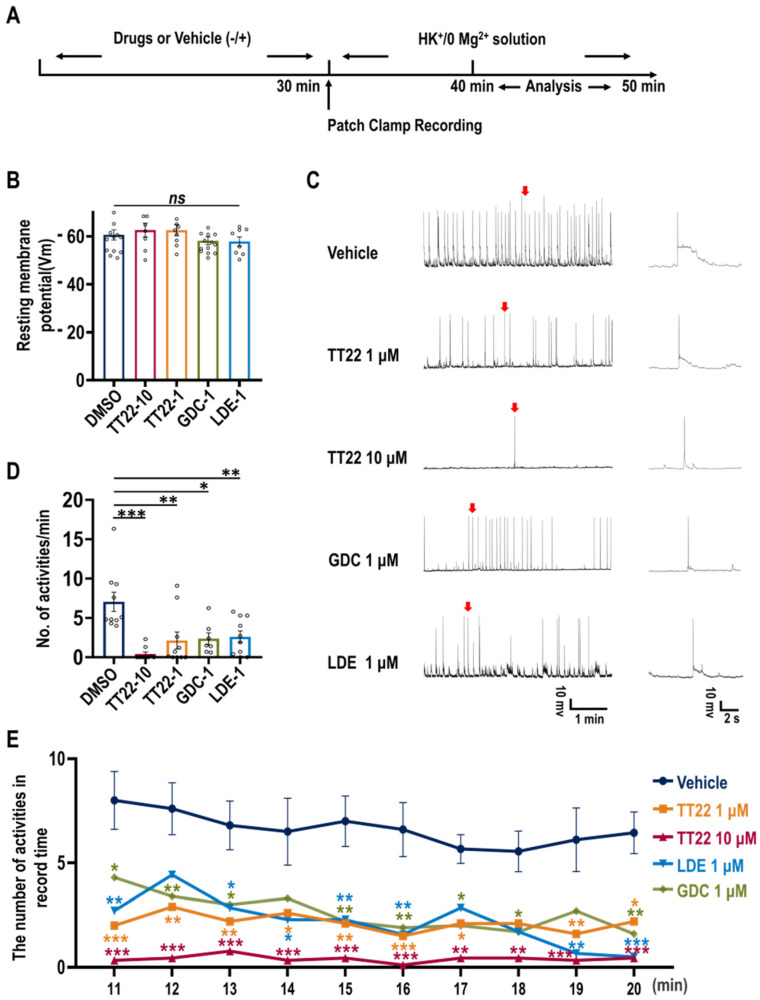
TT22 attenuates the seizure-like activity. (**A**) The cultured hippocampus neurons (*n* = 7–10) were treated with DMSO, TT22, GDC-0449, and LDE-225 for 30 min at 37 °C. Then, neurons were transferred to the patch clamp platform and perfused with high K^+^/0Mg^2+^ extracellular solution without drugs to induce the seizure-like activity until the cells were completely incubated. After that the cells were recorded by patch clamp for 30 min. The recorded data between the 10th and 20th minute were taken for analysis. (**B**) Measurement of resting membrane potential of neurons. After the incubation of indicated drugs for 30 min, neurons were transferred to a standard extracellular solution for whole-cell recordings treated with indicated compounds. Resting membrane potential (Vm) was determined when I = 0. (**C**) Representative traces show the activity of neurons under the indicated treatment and the expanded view of a single burst (arrow). (**D**) The firing frequency of neurons were quantitated. (**E**) The number of seizure-like activities between the 10th and 20th minute was quantitated. All data are means ± SEM (one-way ANOVA and two-way ANOVA). * *p* < 0.05 ** *p* < 0.01 *** *p* < 0.001.

To evaluate the effect of Smo inhibitors on electrophysiological responses of neurons, we used proconvulsant solution containing Mg^2+^-free/K^+^-high (0 Mg^2+^/8 K^+^) to record seizure-like activity. The experiment scheme is shown in Figure 9A. The treatment of TT22, GDC-0449, and LDE-225 did not affect the resting membrane potential of neurons as shown (Figure 9B). Neurons treated with indicated small molecules and the seizure-like activity are measured using methods as described in the Materials and Methods section. TT22, GDC-0449, and LDE-225 inhibited the seizure-like activity (Figure 9C). TT22 as well as GDC-0449 and LDE-225 can reduce the frequency of seizure-like activity (Figure 9D). Furthermore, the recorded data of 10–20 min drug treatment showed that TT22 with a concentration of 1 μM or 10 μM as well as GDC-0449, and LDE-225 treatment inhibited the seizure-like activity (Figure 9E). These results indicate that TT22, GDC-0449, and LDE-225 could effectively inhibit abnormal seizure-like activity in neurons by suppressing Shh signaling.

## 3. Discussion 

Epilepsy is one of the common chronic recurrent diseases of the brain caused by highly synchronized discharges of neurons [30]. The World Health Organization reports that there are about 70 million patients with epilepsy all over the world [13]. Most of antiseizure medications (ASMs) are mainly anticonvulsant, which directly act on the ion channel to rapidly reduce the excitability of the neurons during attack to control the seizure [31]. ASMs widely used are phenobarbital, phenytoin, and ethosuximide which belong to three categories through inhibiting high-frequency neuronal discharges by blocking voltage-dependent sodium channels, blocking T-type calcium channels (T-type VGCC), and enhancing γ-aminobutyric acid type A receptor (GABA_A_ receptor) mediated postsynaptic inhibition [32]. However, about 30% of patients are insensitive to the existing drug treatment, which is referred to as refractory epilepsy. Only surgery is suitable for refractory epilepsy patients [14]. There is a medical need for the treatment of refractory epilepsy, and finding a new treatment for it is still a challenge. 

Shh signaling is important for brain development and homeostasis. Its activity is abnormally increased in advanced BCC, MB, and other cancers as well as refractory temporal lobe epilepsy patients [33,34]. For patients with refractory epilepsy, it has been reported that Shh expression increased in the epileptogenic zone [20]. The abnormal increase of neuronal activity leads to the release of Shh, and regulates glutamate-mediated functions which may contribute to the occurrence and development of refractory epilepsy [24]. These data indicate that Smo in Shh signaling could be a new therapeutic target for refractory epilepsy.

We discovered that the small molecule TT22 obtained from the high-throughput screening can inhibit Shh signaling. Furthermore, TT22, GDC-0449, and LDE-225 can effectively inhibit seizure-like activity in neurons and significantly reduce the frequency and increase the interictal stage. It has been reported that the effects of Smo inhibitors on inhibition abnormal epileptiform discharge in neurons within 30 min of compound treatments suggests that this process is mediated by noncanonical Shh signaling [35,36]. In the postnatal rodent hippocampus, the noncanonical Shh signaling plays an important role during early postnatal neuronal circuit construction and synaptic plasticity involving intracellular Ca^2+^ signaling and the BDNF-TrkB signaling pathway as well as has a role in regulating GABAergic transmission [37,38]. The underlying mechanisms will be intriguing to study in the future.

Previously we have identified 0025A as a novel Smo inhibitor. The IC50 of TT22 and 0025A are 4.75 nM and 1.7 nM, respectively. However, the TT22 displays more activities for the inhibition of abnormal seizure-like activity in neurons. Further studies of the structure–activity relationship (SAR) analysis are needed to understand the mechanism of action of drugs (MOA) for clinical development between these two compounds. In addition, the therapeutic effect of TT22 as well as GDC-0449 and LDE-225 on epilepsy should be further studied in vivo to explore whether these inhibitors decrease epilepsy symptoms. Our study provides a potential new treatment by targeting the Shh signaling pathway associated with refractory epilepsy with a novel Smo inhibitor TT22 which can effectively inhibit seizure-like activity in cultured neurons. We will evaluate the effect of TT22 on the epileptiform discharges and epileptic activities in vitro and on animal models in future studies. Overall, TT22 as well as FDA-approved Smo inhibitors (GDC-0449 and LDE-225) could be developed as new antiseizure medications through targeting Shh signaling pathways associated with refractory epilepsy. These studies could provide a basis for further clinical trials and may provide a new therapy for refractory epilepsy.

**Figure 10:**
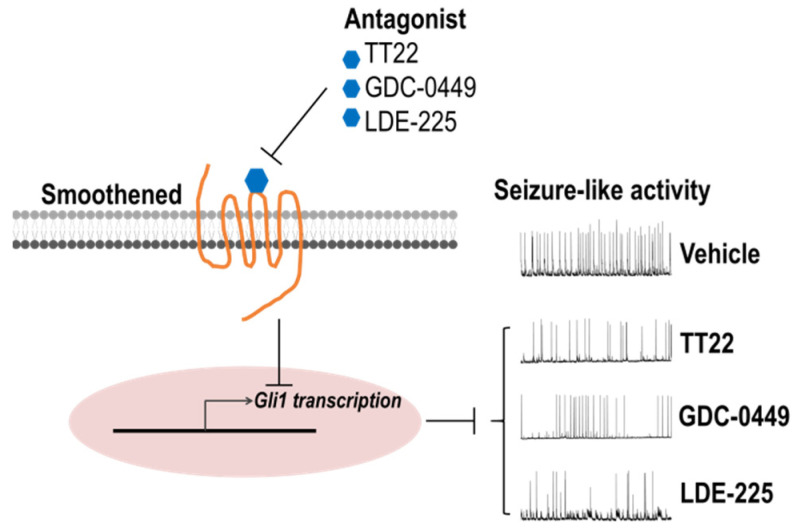
Scheme of the inhibition of Smoothened antagonists on seizure-like activity. Antagonists TT22, GDC-0449, and LDE-225 can bind with a Smoothened receptor, repress its activity, and then inhibit the expression of a downstream Gli1 transcription factor, thus suppressing the abnormal seizure-like activity in hippocampal neurons.

In summary, we identify TT22 as a novel antagonist of Smo. Most importantly, TT22 as well as GDC-0449 and LDE-225 effectively inhibit seizure-like activity (Figure 10). These findings provide a new avenue for targeting Shh signaling associated refractory epilepsy and lay out a basis for future clinical trials.

## 4. Materials and Methods

### 4.1. Chemicals

SAG was generated by YuezhiKangtai Biomedicines (Beijing, China). TT22 was synthesized by Bellen. Bodipy-cyclopamine was obtained from Biovision. Cyclopamine, LDE-225, and GDC-0449 were purchased from Cayman.

### 4.2. N-Shh Conditioned Medium 

The pRK5-ShhN plasmid was transfected into 293FT cells using Lipofectamine 3000 (Invitrogen, Carlsbad, CA, USA). Twenty-four hours later, the medium was replaced with fresh DMEM supplemented with 2% FBS. The culture medium was harvested at 24 h and 48 h after the medium change, then combined and centrifuged for 10 min at 1000 rpm. The supernatant was considered as N-Shh conditioned medium (Shh-CM) and stored at −20 °C until used.

### 4.3. Cell Culture, Plasmids, and Western Blotting

The 293FT, NIH3T3 and U2OS cells were obtained from Dr. Zhiheng Xu. The PTCH−/− MEF cells were provided by Dr. Steven Y. Cheng. The U2OS, NIH3T3, 293FT and PTCH−/− MEF cells were cultured in DMEM (Gibco, Waltham, MA, USA) containing a 10% fetal bovine serum (Hyclone, Logan, UT, USA). To detect the effect of TT22 on Gli1 expression, NIH3T3 cells were seeded into a 12-well plate at the density of 2 × 10^5^ per well and cultured at 37 °C. When the cells were at 100% confluence, they were starved in DMEM supplemented with 0.5% FBS for 1 h at 37 ℃, then treated with 20% Shh-CM or 100 nM SAG with different concentrations of indicated compounds for 24 h at 37 °C. After treatment, the cells were lysed by RIPA, and centrifuged at 12,000 rpm for 10 min at 4 °C. Cell lysates were harvested and detected by Western blotting with the following primary antibodies: Gli1 (CST, 2534s, 1:1000, Boston, MA, USA); and Tubulin (CST, 3873 s, 1:2000).

### 4.4. Immunofluorescence Staining

For cell staining, Ptch^−/−^ MEF cells were cultured and seeded on coverslips. Then, 4% (*w*/*v*) paraformaldehyde/phosphate-buffered saline (PBS) was used to fix cells at room temperature for 20 min. After fixation, the cells were washed three times with PBS, blocked with 5% (*w*/*v*) bovine serum albumin (BSA)/PBS at room temperature for 1 h, and incubated with a primary antibody in 5% BSA overnight at 4 °C. Then, the cells were washed and treated with secondary antibodies conjugated with Alexa Fluor 488 or 568 dyes in 5% BSA at room temperature for 1 h. The images were achieved through LSM710 Zeiss confocal microscope and analyzed with Image J software. The following primary antibodies were applied for immunostaining: ARL 13B (Proteintech, Wuhan, China, 17711-1-AP, 1:100); and Smoothened (Santa Cruz, CA, USA, sc-166685, 1:100).

### 4.5. Primary Rat Hippocampal Neuron Culture

The hippocampus of an SD rat brain at around embryonic day 18 was dissected and digested with 0.05% trypsin containing Dnase I (1:200) for 5–10 min. After terminating the digestion, the cell suspension was gently pipetted to single cell as soon as possible, filtered with a 70-mesh cell sieve, and centrifuged at 1000 rpm for 4 min. Then, fresh DMEM medium was added to resuspend the cells and mixed by gentle pipetting. After counting, suspended cells were seeded at a density of 5 × 10^4^/cm^2^ onto coverslips (Fisherbrand, Waltham, MA, USA) which had been precoated by poly-D-lysine (20 μg/mL, Sigma-Aldrich, St. Louis, MO, USA) at room temperature for 1 h, and cultured in neurobasal medium supplemented with 10% B-27 and 0.5 mM gluta-max at 37 °C for 10–14 days before use.

### 4.6. RNA Isolation, Reverse Transcription, and Real-Time PCR

Total RNA was isolated using the TRIzol reagent (Thermo, Waltham, MA, USA) according to the manufacturer’s recommended procedures, and reverse transcription was performed with PrimeScript™ RT reagent Kit (TaKaRa, Osaka, Japan). Real-time PCR was carried out according to the instructions of the manufacturer. The expression of Gli1 mRNA level was normalized to Actin and determined by real-time PCR according to the 2^−∆∆Ct^ method. The primers used to amplify specific regions for real-time PCR were mouse Gli1, F: 5′-CTCAAACTGCCCAGCTTA ACCC-3′, R: 5′-TGCGGCTGACTGTGTAAGCAGA-3′; mouse Actin, F: 5′-GCAAGTGCTTCTAGGCGGAC-3′, and R: 5′-AAGAAAGGGTGTAAAACGCAG C-3′.

### 4.7. Bodipy-Cyclopamine Binding Assay

Human flag-tagged Smo WT was transfected into HEK293 cells. The transfected cells were digested by trypsin 24 h later and washed in phenol-red free DMEM containing 0.5% FBS. Then, 4% (*w*/*v*) paraformaldehyde/PBS was used to fix cells at room temperature for 10 min. After fixation, the cells were washed with phenol-red free DMEM containing 0.5% FBS and incubated with 5 nM Bodipy-cyclopamine and a range of concentration (10^−9^–10^−5^ M) of indicated compounds at 37 °C for 2 h. Following incubation, the cells were washed, and the fluorescent signals analyzed by flow cytometry.

### 4.8. Hippocampal Neuron Culture Electrophysiology

Whole-cell recordings were performed on primary neurons incubated with different doses of drugs prior to recording in parallel on the same day (day 10–14 in vitro) viewed with an infrared differential interference contrast microscope with an Olympus 40 × water immersion lens (BX51WI, Olympus, Tokyo, Japan) at room temperature (23–24 °C). All recordings were acquired with patch-clamp amplifiers (MultiClamp 700B; Molecular Devices) under the control of pClamp Clampex 10.3 software (Molecular Devices, Rochester, NY, USA). Data were acquired at 10 kHz and low-pass filtered at 1 kHz. Recording electrodes (resistance, 3–6 MΩ) were prepared from borosilicate glass capillaries (BF150-86-75, Sutter Instruments, USA) using a vertical pipette puller (PC-100, Narishige, Chengmao, Japan). The pipette solution contained: K-gluconate 120 mM, KCl 20 mM, HEPES 10 mM, EGTA 10 mM, MgCl_2_ 2 mM, and Na_2_ATP 2 mM (pH 7.3 adjusted with KOH; osmolarity maintained at 300 mOsm). Standard extracellular solution contained the following: NaCl 140 mM, KCl 2.4 mM, HEPES 10 mM, glucose 10 mM, MgCl_2_ 4 mM, and CaCl_2_ 2 mM (pH 7.35 adjusted with NaOH, 310 mOsm). 

Resting membrane potential (V_m_) was determined when I = 0. The proconvulsant solution containing Mg^2+^-free/ K^+^-high (0 Mg^2+^/8 K^+^) was used to record seizure-like activity [39,40,41]. In the current-mode, continuous recordings were performed for 30 min. The seizure-like activity was divided into two forms: the first was a large depolarization shift with ≥10 mV depolarization and ≥300 ms in duration and at least five action potentials; the second was an action potential featured with a significant increase in amplitude above the baseline and high frequency of signals. We carried out the events described, as above, when the cell attained the stable epileptiform state between 10 and 20 min after we had transferred the cells into proconvulsant solution. Cohorts of cells included in the “Vehicle” group were pretreated with an equal dose of vehicle solution (dimethyl sulfoxide, DMSO), while the “Drug” groups were subjected to different drugs which needed to be tested with varied concentrations for 30 min prior to patch recording.

### 4.9. Statistical Analysis

All data were acquired from more than two independent experiments and analyzed using Prism software (GraphPad Software, Inc., San Diego, CA, USA). An unpaired Student’s t test was performed for two-sample comparisons. One-way ANOVA was applied for multiple comparisons, followed by a p value adjustment with the Bonferroni method, and a two-way ANOVA was performed for multiple comparisons with two independent variables. All values were expressed as means ± SEM, and * *p* < 0.05 was judged to be statistically significant. 

## 5. Conclusions

The new Smoothened inhibitor TT22 as well as the GDC-0449 and LDE-225 may provide a new therapy targeting sonic hedgehog signaling for refractory epilepsy.

## Data Availability

The data presented in this article are available.

## References

[1] Gorojankina T. (2016). Hedgehog signaling pathway: A novel model and molecular mechanisms of signal transduction. Cell. Mol. Life Sci..

[2] Chen W., Ren X.R., Nelson C.D., Barak L.S., Chen J.K., Beachy P.A., de Sauvage F., Lefkowitz R.J. (2004). Activity-dependent internalization of smoothened mediated by beta-arrestin 2 and GRK2. Science.

[3] Robbins D.J., Fei D.L., Riobo N.A. (2012). The Hedgehog signal transduction network. Sci. Signal..

[4] Arensdorf A.M., Marada S., Ogden S.K. (2016). Smoothened Regulation: A Tale of Two Signals. Trends Pharmacol. Sci..

[5] Yang L., Xie G., Fan Q., Xie J. (2010). Activation of the hedgehog-signaling pathway in human cancer and the clinical implications. Oncogene.

[6] Wu F., Zhang C., Zhao C., Wu H., Teng Z., Jiang T., Wang Y. (2020). Prostaglandin E1 Inhibits GLI2 Amplification-Associated Activation of the Hedgehog Pathway and Drug Refractory Tumor Growth. Cancer Res..

[7] Robinson G.W., Orr B.A., Wu G., Gururangan S., Lin T., Qaddoumi I., Packer R.J., Goldman S., Prados M.D., Desjardins A. (2015). Vismodegib Exerts Targeted Efficacy against Recurrent Sonic Hedgehog-Subgroup Medulloblastoma: Results from Phase II Pediatric Brain Tumor Consortium Studies PBTC-025B and PBTC-032. J. Clin. Oncol..

[8] Pan S., Wu X., Jiang J., Gao W., Wan Y., Cheng D., Han D., Liu J., Englund N.P., Wang Y. (2010). Discovery of NVP-LDE225, a Potent and Selective Smoothened Antagonist. ACS Med. Chem. Lett..

[9] Li Y., Song Q., Day B.W. (2019). Phase I and phase II sonidegib and vismodegib clinical trials for the treatment of paediatric and adult MB patients: A systemic review and meta-analysis. Acta Neuropathol. Commun..

[10] Wang J., Lu J., Bond M.C., Chen M., Ren X.R., Lyerly H.K., Barak L.S., Chen W. (2010). Identification of select glucocorticoids as Smoothened agonists: Potential utility for regenerative medicine. Proc. Natl. Acad. Sci. USA.

[11] Wang J., Mook R.A., Lu J., Gooden D.M., Ribeiro A., Guo A., Barak L.S., Lyerly H.K., Chen W. (2012). Identification of a novel Smoothened antagonist that potently suppresses Hedgehog signaling. Bioorg. Med. Chem..

[12] Fan J., Li H., Kuang L., Zhao Z., He W., Liu C., Wang Y., Cheng S.Y., Chen W. (2021). Identification of a potent antagonist of smoothened in hedgehog signaling. Cell Biosci..

[13] Fisher R.S., Acevedo C., Arzimanoglou A., Bogacz A., Cross J.H., Elger C.E., Engel J., Forsgren L., French J.A., Glynn M. (2014). ILAE official report: A practical clinical definition of epilepsy. Epilepsia.

[14] Thijs R.D., Surges R., O’Brien T.J., Sander J.W. (2019). Epilepsy in adults. Lancet.

[15] Perucca P., Gilliam F.G. (2012). Adverse effects of antiepileptic drugs. Lancet Neurol..

[16] Delorenzo R.J., Sun D.A., Deshpande L.S. (2005). Cellular mechanisms underlying acquired epilepsy: The calcium hypothesis of the induction and maintainance of epilepsy. Pharmacol. Ther..

[17] Brodie M.J., Dichter M.A. (1996). Antiepileptic drugs. N. Engl. J. Med..

[18] Engel J., McDermott M.P., Wiebe S., Langfitt J.T., Stern J.M., Dewar S., Sperling M.R., Gardiner I., Erba G., Fried I. (2012). Early surgical therapy for drug-resistant temporal lobe epilepsy: A randomized trial. JAMA.

[19] Ramos-Perdigues S., Bailles E., Mane A., Carreno M., Donaire A., Rumia J., Bargallo N., Boget T., Setoain X., Valdes M. (2018). Psychiatric Symptoms in Refractory Epilepsy during the First Year After Surgery. Neurotherapeutics.

[20] Fang M., Lu Y., Chen G.J., Shen L., Pan Y.M., Wang X.F. (2011). Increased expression of sonic hedgehog in temporal lobe epileptic foci in humans and experimental rats. Neuroscience.

[21] Patel S.S., Tomar S., Sharma D., Mahindroo N., Udayabanu M. (2017). Targeting sonic hedgehog signaling in neurological disorders. Neurosci. Biobehav. Rev..

[22] Li X., Li Y., Li S., Li H., Yang C., Lin J. (2021). The role of Shh signalling pathway in central nervous system development and related diseases. Cell Biochem. Funct..

[23] Xie Y.J., Zhou L., Jiang N., Zhang N., Zou N., Zhou L., Wang Y., Cowell J.K., Shen Y. (2015). Essential roles of leucine-rich glioma inactivated 1 in the development of embryonic and postnatal cerebellum. Sci. Rep..

[24] Feng S., Ma S., Jia C., Su Y., Yang S., Zhou K., Liu Y., Cheng J., Lu D., Fan L. (2016). Sonic hedgehog is a regulator of extracellular glutamate levels and epilepsy. EMBO Rep..

[25] Chen J.K., Taipale J., Young K.E., Maiti T., Beachy P.A. (2002). Small molecule modulation of Smoothened activity. Proc. Natl. Acad. Sci. USA.

[26] Wang Y., Zhou Z., Walsh C.T., McMahon A.P. (2009). Selective translocation of intracellular Smoothened to the primary cilium in response to Hedgehog pathway modulation. Proc. Natl. Acad. Sci. USA.

[27] Wang Y., Arvanites A.C., Davidow L., Blanchard J., Lam K., Yoo J.W., Coy S., Rubin L.L., McMahon A.P. (2012). Selective identification of hedgehog pathway antagonists by direct analysis of smoothened ciliary translocation. ACS Chem. Biol..

[28] Wu V.M., Chen S.C., Arkin M.R., Reiter J.F. (2012). Small molecule inhibitors of Smoothened ciliary localization and ciliogenesis. Proc. Natl. Acad. Sci. USA.

[29] Osterlund T., Kogerman P. (2006). Hedgehog signalling: How to get from Smo to Ci and Gli. Trends Cell Biol..

[30] Scheffer I.E., Berkovic S., Capovilla G., Connolly M.B., French J., Guilhoto L., Hirsch E., Jain S., Mathern G.W., Moshe S.L. (2017). ILAE classification of the epilepsies: Position paper of the ILAE Commission for Classification and Terminology. Epilepsia.

[31] Schmidt D., Schachter S.C. (2014). Drug treatment of epilepsy in adults. BMJ.

[32] Rogawski M.A., Loscher W., Rho J.M. (2016). Mechanisms of Action of Antiseizure Drugs and the Ketogenic Diet. Cold Spring Harb. Perspect. Med..

[33] Thompson M.C., Fuller C., Hogg T.L., Dalton J., Finkelstein D., Lau C.C., Chintagumpala M., Adesina A., Ashley D.M., Kellie S.J. (2006). Genomics identifies medulloblastoma subgroups that are enriched for specific genetic alterations. J. Clin. Oncol..

[34] Kool M., Jones D.T., Jager N., Northcott P.A., Pugh T.J., Hovestadt V., Piro R.M., Esparza L.A., Markant S.L., Remke M. (2014). Genome sequencing of SHH medulloblastoma predicts genotype-related response to smoothened inhibition. Cancer Cell.

[35] Belgacem Y.H., Borodinsky L.N. (2011). Sonic hedgehog signaling is decoded by calcium spike activity in the developing spinal cord. Proc. Natl. Acad. Sci. USA.

[36] Belgacem Y.H., Borodinsky L.N. (2015). Inversion of Sonic hedgehog action on its canonical pathway by electrical activity. Proc. Natl. Acad. Sci. USA.

[37] Delmotte Q., Diabira D., Belaidouni Y., Hamze M., Kochmann M., Montheil A., Gaiarsa J.L., Porcher C., Belgacem Y.H. (2020). Sonic Hedgehog Signaling Agonist (SAG) Triggers BDNF Secretion and Promotes the Maturation of GABAergic Networks in the Postnatal Rat Hippocampus. Front Cell. Neurosci..

[38] Delmotte Q., Hamze M., Medina I. (2020). Smoothened receptor signaling regulates the developmental shift of GABA polarity in rat somatosensory cortex. J. Cell Sci..

[39] Zhang Y., Huang Y., Liu X., Wang G., Wang X., Wang Y. (2015). Estrogen suppresses epileptiform activity by enhancing Kv4.2-mediated transient outward potassium currents in primary hippocampal neurons. Int. J. Mol. Med..

[40] Stanton P.K., Jones R.S., Mody I., Heinemann U. (1987). Epileptiform activity induced by lowering extracellular [Mg2+] in combined hippocampal-entorhinal cortex slices: Modulation by receptors for norepinephrine and N-methyl-D-aspartate. Epilepsy Res..

[41] Solger J., Heinemann U., Behr J. (2005). Electrical and chemical long-term depression do not attenuate low-Mg2+-induced epileptiform activity in the entorhinal cortex. Epilepsia.

